# ILK Deletion Protects Against Chronic Kidney Disease-Associated Vascular Damage

**DOI:** 10.3390/ijms27010215

**Published:** 2025-12-24

**Authors:** Sofía Campillo, Elena Gutiérrez-Calabrés, Susana García-Miranda, Mercedes Griera, Sergio de Frutos, Diego Rodríguez-Puyol, Laura Calleros

**Affiliations:** 1 Physiology Unit, Department of Systems Biology, Universidad de Alcalá, 28871 Alcalá de Henares, Madrid, Spain; elena.gutierrez@uah.es (E.G.-C.); susana.garciam@uah.es (S.G.-M.); mercedes.griera@uah.es (M.G.); sergio.frutos@uah.es (S.d.F.); laura.calleros@uah.es (L.C.); 2 Fundación Renal Española, 28003 Madrid, Spain; diego.rodriguez@uah.es; 3 Instituto Ramón y Cajal de Investigación Sanitaria (IRYCIS), 28034 Madrid, Spain; 4 INNOREN-CM, 28049 Madrid, Spain; 5 RICORS 2040 RENAL, Instituto de Salud Carlos III, 28029 Madrid, Spain; 6 Department of Medicine and Medical Specialties, Universidad de Alcalá, 28871 Alcalá de Henares, Madrid, Spain; 7 Biomedical Research Foundation and Nephrology Unit, Hospital Universitario Príncipe de Asturias, 28805 Alcalá de Henares, Madrid, Spain

**Keywords:** ILK, fibrosis, inflammation, vascular damage, chronic kidney disease (CKD)

## Abstract

Cardiovascular diseases are a major cause of morbidity and mortality in chronic kidney disease (CKD) patients. Integrin-linked kinase (ILK) regulates integrin–extracellular matrix interactions and vascular integrity. This study investigated the role of ILK in CKD-associated vascular alterations. An adenine-supplemented diet induced a progressive CKD in wild-type (WT) and conditional ILK knock-down (cKD-ILK) mice. Aortic tissue was collected for histology and RT-qPCR analysis. Moreover, aortas were incubated ex vivo with the uremic toxins *p*-cresyl sulfate and indoxyl sulfate. In vitro, human aortic vascular smooth muscle cells were exposed to uremic toxins, and the effect of siRNA-mediated ILK silencing was tested. Aortas of adenine-fed WT mice showed a progressive increase in ILK expression, morphological alterations, and increased fibrosis, which was not observed in cKD-ILK aortas, compared to control mice. Statistically significant correlations between vascular content of ILK and fibrosis markers were observed. Ex vivo, uremic toxins increased ILK and fibrosis protein expression in WT aortas but not in cKD-ILK. In vitro, uremic toxins increased ILK activity and fibrosis markers, like collagen, while ILK-deleted cells prevented collagen increase. ILK depletion prevents CKD-associated vascular fibrosis, suggesting ILK as a potential therapeutic target to prevent arterial alterations in renal patients.

## 1. Introduction

Nowadays, the direct relationship between chronic kidney disease (CKD) and cardiovascular diseases (CVDs) is indisputable. Patients with CKD have a higher risk of developing CVD, such as ischemic heart disease, cerebrovascular disease, peripheral vascular disease, and heart failure, than the general population [1]. In fact, high cardiovascular mortality prevents most patients with CKD from progressing to End-Stage Renal Disease (ESRD), which means that CKD directly impacts the global burden of mortality caused by CVD, the most common cause of premature morbidity and mortality worldwide [2]. Therefore, early identification and treatment of cardiovascular damage in patients with CKD can reduce the severity of the disease and improve the outcomes of those who reach renal replacement therapy [3].

Patients with CKD show alterations that affect vascular functionality, such as abnormal endothelium-dependent vasodilatation, increased oxidative stress, alterations in antithrombotic and anti-inflammatory properties, atheroma plaques, vascular calcifications, and arterial stiffness [4,5]. Apart from traditional cardiovascular risk factors (advanced age, hypertension, hyperlipidemia or diabetes), patients with CKD are conditioned by other non-traditional factors, including oxidative stress, inflammation or the accumulation of various uremic toxins, which are largely responsible for the severity and specificity of the circulatory damages that accompany CKD.

In recent years, much attention has been focused on the metabolic products of the intestinal microbiota as a source of many uremic toxins [6]. In CKD, the intestinal barrier is altered, leading to increased production of uremic toxins derived from the metabolism of intestinal bacteria [7]. Within the uremic toxins, *p*-cresyl sulfate (pCS) and indoxyl sulfate (IS) are protein-bound uremic toxins that cannot be easily removed by conventional dialysis methods because of their high binding affinity for albumin [8]. At the cardiovascular level, pCS and IS have been identified as pathogenic agents associated with cardiovascular mortality in patients with CKD [9,10,11,12].

Vascular development and maintenance have been shown to be regulated by integrin-extracellular matrix (ECM) interactions and endothelial and vascular cell survival, where integrin-linked kinase (ILK) plays a key role [13,14,15]. ILK is a serine/threonine protein kinase and a scaffold protein which acts by regulating signal transduction from the extracellular medium to the cellular interior and vice versa [16,17]. Numerous investigations have shown that ILK is essential for both the maintenance of vascular and endothelial integrity, and the regulation of leukocyte recruitment and adhesion to the vascular endothelium after tissue damage, including in a CKD context [17,18,19,20,21,22,23]. However, the role of ILK in the vascular smooth muscle alterations derived from CKD is unknown.

This study aimed to investigate the involvement of ILK in vascular damage associated with CKD. To this end, arterial alterations were evaluated using an experimental model of CKD induced by adenine and human vascular smooth muscle cells treated with uremic toxins.

## 2. Results

### 2.1. ILK Content Is Increased in the Aortas of Mice with Experimental CKD

We analyzed the renal function of WT and cKD-ILK mice fed a standard or adenine-rich diet for 2, 4, or 6 weeks. The progressive decrease in glomerular filtration rate from the 2nd to the 6th week of adenine diet intake, determined by the increase in urea nitrogen and creatinine plasma concentrations, was previously published by our group [2,4] and is shown in Table 1. In contrast, adenine-fed cKD-ILK mice showed decreased urea nitrogen and creatinine plasma concentrations compared to adenine-fed WT mice from week 2 onwards, although these values did not fully return to those observed in WT control animals.

**Figure 1:**
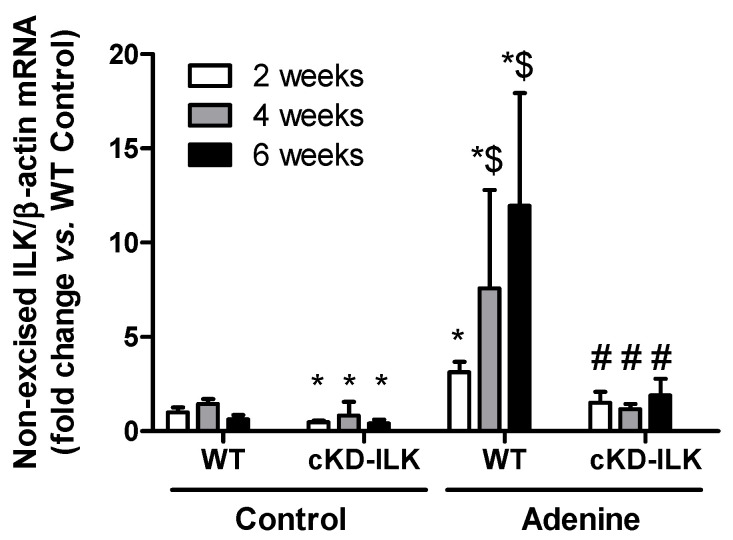
ILK content increases progressively in the aortas of adenine-fed mice. Wild-type (WT) and ILK conditional-knockdown (cKD-ILK) mice were fed a standard (Control) or an adenine-rich (Adenine) diet for 2, 4 or 6 weeks. Non-excised ILK mRNA expression in the aortas, normalized against β-actin as the endogenous control, was measured. Results were expressed as percentage with respect to WT Control mice. Results are shown as mean ± SEM. * *p* < 0.05 vs. WT Control at the same time; ^$^ *p* < 0.05 vs. 2 weeks; ^#^ *p* < 0.05 vs. WT Adenine at the same time. n = 3–9 animals/group.

Then, we analyzed the expression of ILK in the aortas of mice. Non-excised ILK mRNA expression in aortic tissues from mice fed the adenine-rich diet, compared to mice fed the standard diet, increased significantly (Figure 1). As shown in this figure, the changes in ILK mRNA expression exhibited a progressively increasing profile in parallel to the progression of CKD, which was statistically significant from the 2nd week of adenine diet intake. ILK transgenic depletion prevented the ILK expression increase in cKD-ILK mice (Figure 1).

### 2.2. Aortas of Mice with Experimental CKD Exhibit Structural Damages That Are Prevented by ILK Deletion

To determine whether systemic changes such as weight loss or hypertension could influence vascular outcomes, we measured body weight and blood pressure in all experimental groups (Table 2). Adenine-fed WT mice showed a progressive reduction in body weight starting from the second week and developed hypertension by the fourth week. Interestingly, as we previously published [24], adenine-fed cKD-ILK mice exhibited lower blood pressure compared to adenine-fed WT mice, although their values did not reach those of the control WT group.

**Figure 2:**
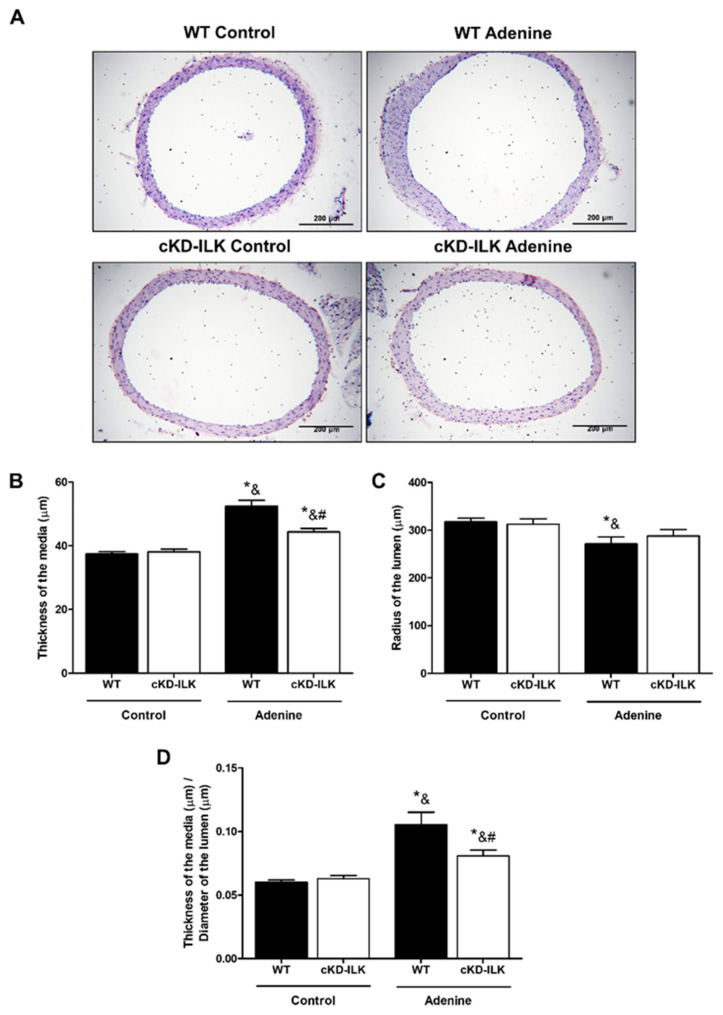
ILK deletion protected against structural vascular damage in adenine-fed mice. Wild-type (WT) and conditional ILK deletion (cKD-ILK) mice were fed a standard (Control) or adenine-rich (Adenine) diet for 6 weeks. Cross sections of aortas were analyzed histologically. (**A**) Representative microphotographs of hematoxylin-eosin (10×) staining are shown. Scale bar, 200 μm. (**B**–**D**) Morphometric analysis was assessed by measuring the thickness of the aorta wall (**B**), the radius of the lumen (**C**), and the ratio between media thickness and lumen diameter of the aortas (**D**). The thickness of the aorta wall and the radius and diameter of the aorta lumen were performed using the ImageJ software. Results are shown as mean ± SEM. * *p* < 0.05 vs. WT Control; ^&^ *p* < 0.05 vs. cKD-ILK Control; ^#^ *p* < 0.05 vs. WT Adenine. n = 5–8 animals/group.

The morphological vascular changes induced by CKD were studied by staining aortas with hematoxylin-eosin (Figure 2A). Aortas from WT mice fed the adenine-rich diet for 6 weeks showed a significant increase in the thickness of the media, a decrease in the radius of the lumen, and an increase in the ratio of the thickness of the media to the diameter of the lumen compared to mice fed the standard diet (Figure 2A–D). In contrast, cKD-ILK animals with CKD showed a smaller increase in both thickness of the media and ratio, whereas no significant decrease in the radius of the lumen was observed with respect to control groups (Figure 2A–D).

**Figure 3:**
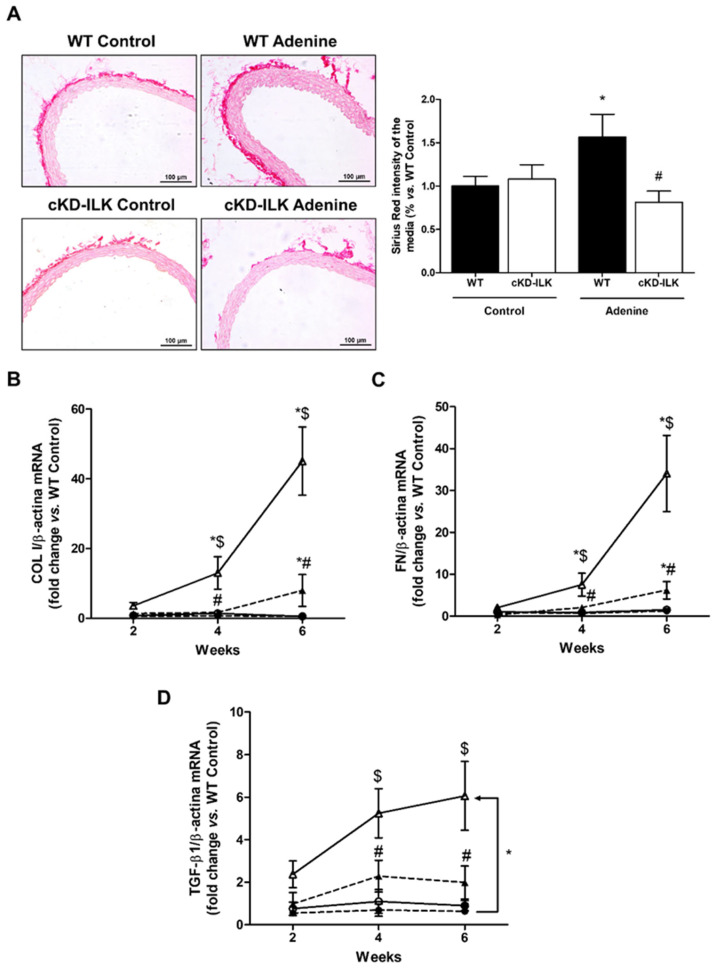
ILK deletion prevented the development of vascular fibrosis in adenine-fed mice. (**A**) Wild-type (WT) and conditional ILK deletion (cKD-ILK) mice were fed a standard (Control) or adenine-rich (Adenine) diet for 6 weeks. Cross sections of aortas were analyzed histologically. Representative microphotographs of Sirius red (20×) staining are shown. Scale bar, 100 μm. Fibrosis in the tunica media of the aorta was determined by the intensity of collagen staining by Sirius red. Results were expressed as a percentage of the intensity of WT Control mice. Results are shown as mean ± SEM. n = 5–8 animals/group. * *p* < 0.05 vs. WT Control; # *p* < 0.05 vs. WT Adenine. (**B**–**D**) Wild-type (WT, solid lines, white symbols) and conditional ILK deletion (cKD-ILK, dashed lines, black symbols) mice were fed a standard (Control, circles) or adenine-rich (Adenine, triangles) diet for 2, 4 or 6 weeks. Collagen I (COL I) (**B**), fibronectin (FN) (**C**) and TGF-β1 (**D**) mRNA expression was measured in aortas, normalized against β-actin as endogenous control. Results are shown as mean ± SEM. n = 3–7 animals/group. * *p* < 0.05 vs. WT Control at the same time; ^$^ *p* < 0.05 vs. 2 weeks; ^#^ *p* < 0.05 vs. WT Adenine at the same time.

**Figure 4:**
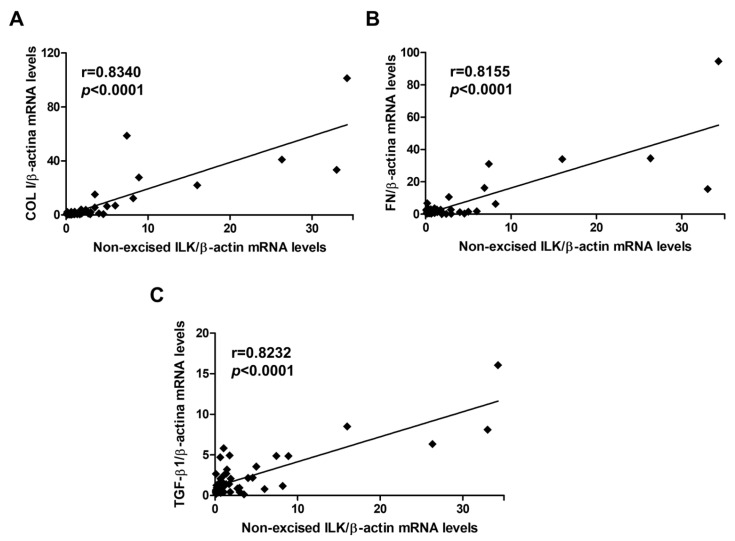
ILK expression correlates with the expression of extracellular matrix proteins, collagen I (COL I) and fibronectin (FN), and TGF-β1 in the aortas. Wild-type and conditional ILK deletion mice were fed a standard or adenine-rich diet for 2, 4 or 6 weeks. ILK mRNA expression was confronted to the values of COL I (**A**), FN (**B**), and TGF-β1 (**C**) mRNA expressions in the aortas. The analysis is detailed in the Materials and methods section. n = 3–7 animals/group.

To determine whether this CKD model also induced fibrosis in vascular tissue, Sirius red staining was performed to assess collagen deposits in the aortic wall. As shown in Figure 3A, WT adenine-fed mice showed a greater presence of collagen deposits in the aortic wall than WT animals, and this increase was not observed in ILK-deleted mice fed the same diet. To confirm the induction of vascular fibrosis in CKD and to determine whether such fibrosis progressed as CKD does, mRNA levels of ECM proteins, such as collagen I and fibronectin, and the profibrotic cytokine TGF-β1 were studied in the aortic tissue of mice with different stages of CKD. WT animals fed the adenine-rich diet presented a progressive increase in the levels of these genes. Such increase was statistically significant from the 4th week of diet intake for collagen I and fibronectin and from the 6th week for TGF-β1 and was decreased significantly in cKD-ILK mice fed the adenine diet (Figure 3B–D). Furthermore, to analyze the physiological relevance of the observed changes in ILK content and fibrosis markers in aortas, these parameters were evaluated simultaneously. These results showed a statistically significant direct correlation between ILK mRNA expression levels and gene expression of collagen I, fibronectin, and TGF-β1 in aortas (Figure 4).

**Figure 5:**
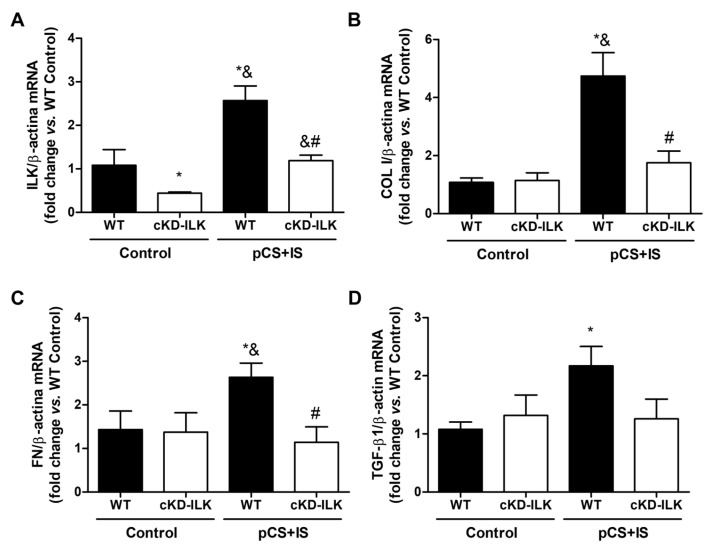
ILK deletion prevented fibrosis marker expression increase in mice aortas treated ex vivo with uremic toxins. Wild-type (WT) and ILK conditional-knockdown (cKD-ILK) mice were fed a standard diet. Aortas were excised and treated ex vivo with a combination of *p*-cresyl sulfate (pCS) and indoxyl sulfate (IS) at high doses (226 µg/mL and 100 µg/mL, respectively) for 24 h. After incubation with the uremic toxins, ILK (**A**), collagen I (COL I) (**B**), fibronectin (FN) (**C**), and TGF-β1 (**D**) mRNA expression in the aortas, normalized against β-actin as an endogenous control, was measured. Results are shown as mean ± SEM. * *p* < 0.05 vs. WT Control; ^&^ p < 0.05 vs. cKD-ILK Control; ^#^ *p* < 0.05 vs. WT (pCS+IS). n = 3–7 animals/group.

However, this evidence alone does not confirm that ILK deletion in vascular tissue protects against CKD-induced structural damage and fibrosis. To rule out that this protection was due to the improvement of renal function conditioned by the ILK deletion at renal level (Table 1), ex vivo experiments were performed. Aortas extracted from WT and cKD-ILK mice were exposed to high doses of the uremic toxins pCS and IS (226 µg/mL and 100 µg/mL, respectively) for 24 h, to simulate uremia, and mRNA levels of ILK and fibrosis markers were analyzed. As shown in Figure 5, uremic toxins induced a significant increase in the expression of all genes compared to untreated aortas from WT mice. In the case of aortas from cKD-ILK mice treated with pCS and IS, mRNA levels of all genes remained close to those of controls (Figure 5). Furthermore, ILK deletion in aortas was again confirmed by decreased ILK expression in vascular tissue of cKD-ILK mice (Figure 5).

### 2.3. Uremic Toxins Increase ILK Activity and Induce Fibrosis in Vascular Smooth Muscle Cells and ILK Deletion Prevents Such Increase

To gain evidence about the role of ILK in CKD-associated vascular damage and to test the possibility that smooth muscle cells could be one of the targets of uremic toxins in the development of vascular fibrosis, we decided to perform in vitro experiments with human aortic vascular smooth muscle cells, HA-VSMC.

First, propidium iodide exclusion viability assays of cells treated with pCS at 226 µg/mL and IS at 100 µg/mL for different times were performed to test whether uremic toxins induced any toxicity on HA-VSMC cells. The exposure of the cells to these toxins did not induce cell death at any time (Supplementary Figure S1), so it was decided to continue with this concentration of uremic toxins for subsequent experiments.

**Figure 6:**
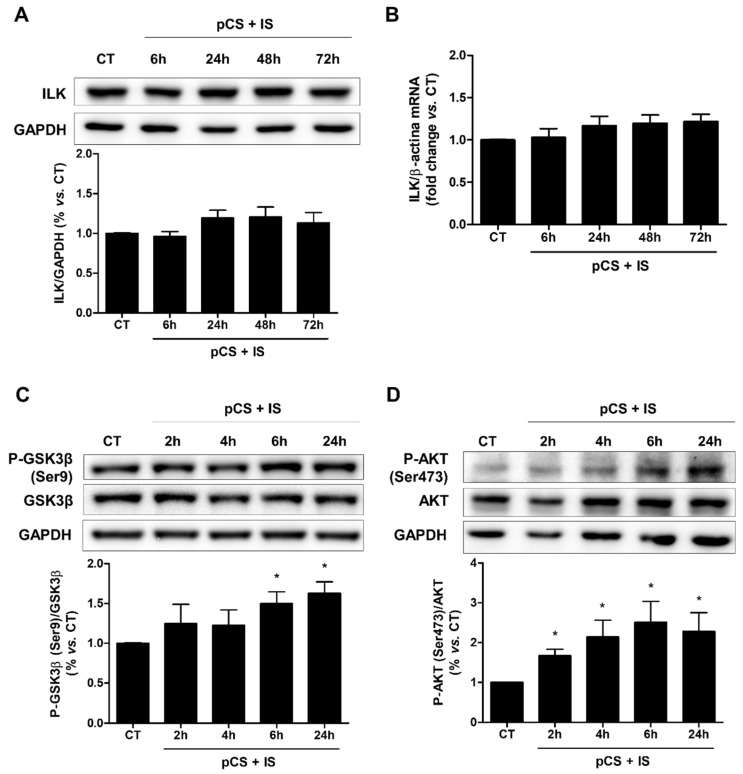
*p*-cresyl sulfate (pCS) and indoxyl sulfate (IS) increase ILK activity and do not regulate its expression in HA-VSMC cells. HA-VSMC cells were incubated with a combination of pCS and IS at high doses (226 µg/mL and 100 µg/mL, respectively) for different times. After incubation with the uremic toxins, ILK expression was measured by Western blot (**A**) and RT-qPCR (**B**) and phosphorylation levels of GSK-3β at serine 9 (P-GSK-3β) (**C**) and AKT at serine 473 (P-AKT) (**D**) were measured by Western blot. (**A**,**C**,**D**) Representative Western blots of ILK, P-GSK-3β, and P-AKT are shown. GAPDH, GSK-3β, and AKT were used as the endogenous controls, respectively. The bars represent the normalized densitometric values of the blots against the endogenous control values. (**B**) ILK mRNA expression was quantified by RT-qPCR. Relative fold changes in mRNA content vs. untreated control (CT) are represented after the normalization with total β-actin content as the endogenous control. All values are presented as the mean ± SEM of 4 independent experiments. * *p* < 0.05 vs. CT.

**Figure 7:**
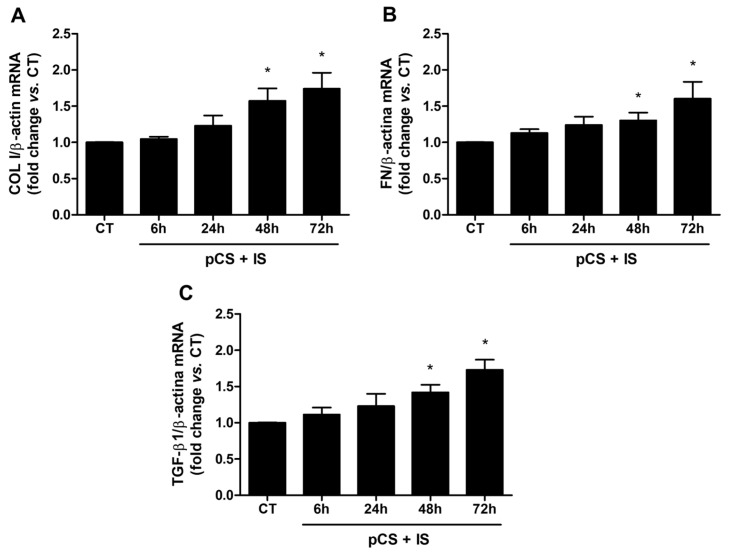
*p*-cresyl sulfate (pCS) and indoxyl sulfate (IS) induce the expression of extracellular matrix proteins and TGF-β1 in HA-VSMC cells. HA-VSMC cells were incubated with a combination of pCS and IS at high doses (226 µg/mL and 100 µg/mL, respectively) for different times. After incubation with the uremic toxins, mRNA expression of collagen I (COL I) (**A**), fibronectin (FN) (**B**), and TGF-β1 (**C**) was measured by RT-qPCR. Relative fold changes in mRNA content vs. untreated control (CT) are represented after the normalization with total β-actin content as the endogenous control. All values are presented as the mean ± SEM of 3 independent experiments. * *p* < 0.05 vs. CT.

Next, the effect of uremic toxins on ILK expression and kinase activity, as determined by increasing the phosphorylation levels of GSK-3β (P-GSK-3β) and AKT (P-AKT), was studied. The combination of both toxins did not affect either the protein content (Figure 6A) or mRNA levels (Figure 6B) of ILK at the concentration of uremic toxins and treatment times used, whereas it did induce rapid and sustained increases over time of P-GSK-3β after 6 h of incubation (Figure 6C) and of P-AKT after 2 h of incubation (Figure 6D). In addition, the expression of markers of fibrosis was analyzed after treatment of cells with pCS and IS. The results showed significant increases in the mRNA levels of collagen I (Figure 7A), fibronectin (Figure 7B), and the profibrotic cytokine TGF-β1 (Figure 7C) compared to untreated cells.

**Figure 8:**
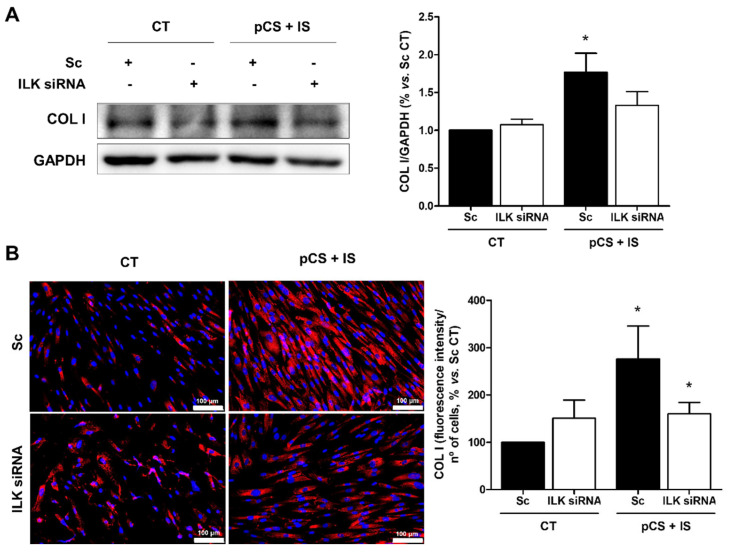
*p*-cresyl sulfate (pCS) and indoxyl sulfate (IS) induce the expression of collagen I (COL I) through ILK in HA-VSMC cells. HA-VSMC cells were transfected with scrambled RNA (Sc, black bars) or were depleted of ILK with specific siRNA (ILK siRNA, white bars) and were incubated with a combination of pCS and IS at high doses (226 µg/mL and 100 µg/mL, respectively) for 48 h. (**A**) After incubation with the uremic toxins, protein expression of COL I was measured by Western blot. Representative Western blots of COL I are shown. GAPDH was used as the endogenous control. The bars represent the normalized densitometric values of the blots against the endogenous control values. (**B**) Prior to transfection, cells were seeded on coverslips and, after incubation with the uremic toxins, cells were labeled with an anti-COL I antibody (red) and nuclei with Hoechst 33342 (blue). Fluorescence intensity was determined by fluorescence microscopy. Images from a representative experiment are shown. Scale bar: 100 μm. The bar graph represents the average percentages of COL I fluorescence intensity normalized with respect to the number of cells in each image. Results were expressed as a percentage with respect to the Sc untreated control (CT). All values are presented as the mean ± SEM of 3 or 4 independent experiments. * *p* < 0.05 vs. Sc CT.

To demonstrate that the increase in GSK-3β phosphorylation induced by pCS and IS in HA-VSMC cells was ILK-dependent and that this could be the mechanism by which fibrosis occurs upon treatment of these cells with uremic toxins, ILK was silenced by specific siRNAs. Firstly, the decrease in ILK protein expression upon silencing with siRNAs was verified, whereas, again, uremic toxins did not modify its expression (Supplementary Figure S2A,B). On the other hand, the increase in GSK-3β phosphorylation induced by uremic toxins was confirmed to be ILK-dependent, as this increase was prevented, at least in part, by silencing ILK (Supplementary Figure S2C). Finally, the involvement of ILK in the development of pCS- and IS-induced fibrosis in HA-VSMC cells was demonstrated by silencing ILK in these cells using specific siRNAs. Treatment of cells with uremic toxins induced a significant increase in protein content of collagen I, whereas silencing ILK in these cells prevented such increase (Figure 8).

## 3. Discussion

In this research, we focus on better understanding the pathogenic processes involved in arterial alterations, which are highly prevalent in CKD, both in an experimental model of CKD and in vascular smooth muscle cells under uremic conditions. The most significant results of this investigation were that ILK expression in aortic tissue increased as CKD progressed and that this increase in ILK content correlated with the expression of different molecules considered markers of aortic damage, whereas its deletion prevented the progression of vascular fibrosis. Moreover, ILK activity seems to be involved in the progression of vascular fibrosis. Therefore, ILK could be a potential therapeutic target for the prevention of arterial alterations produced during CKD.

For this investigation, we studied aortas from an experimental model based on the transgenic deletion of ILK before the induction of CKD, which was generated by administration of high amounts of adenine in the diet for 2, 4, or 6 weeks. Conditional deletion of ILK allowed us to evaluate the involvement of ILK in the progression of vascular damage associated with CKD and the consequences of its deletion in the experimental model of progressive CKD [24]. Adenine metabolism produces a precipitate that occludes renal tubules and damages the tubular epithelium as it results in the formation of intratubular casts, tubular dilatation, inflammation, necrosis, foreign body granulomas, and ultimately tubulointerstitial fibrosis, leading to renal dysfunction characterized by elevated serum urea and creatinine levels [25,26,27]. Ultimately, adenine induces a tubulointerstitial damage profile similar to that seen in human CKD. Depending on the research aim, the adenine CKD model has been tested at different times (from 2 to 8 weeks of diet) [28,29,30]. Since adenine induces progressive and gradual renal damage [24], we decided to choose three treatment times (2, 4, and 6 weeks) that would allow us to evaluate the consequences of ILK deletion on aortic tissue alterations from the onset and throughout the entire progression of CKD, up to the ESRD.

Our results showed that ILK expression in mouse aortas gradually increased as CKD progressed, whereas no such increase was observed in mice with ILK deletion, as we have already described in renal tissue [24]. This finding suggests that ILK could play an important role from the origin and throughout the progression of the arterial alterations produced during CKD. In fact, numerous investigations have studied the function of ILK in physiological contexts, such as vascular development [13,14,15], as well as in pathophysiological contexts, such as the maintenance of vascular and endothelial integrity [18,19], vascular tone [31,32,33] and the regulation of ECM proliferation, migration, and synthesis in smooth muscle cells [34], processes essential for the repair of lesions in vascular pathologies, such as atherosclerosis and restenosis.

In the aortas of mice fed the adenine-rich diet we observed structural abnormalities, such as increased media thickness and number of collagen fibers and decreased lumen radius, and increases in the expression of markers of fibrosis in the aortas. These results agreed with other studies performed in experimental models and in patients in whom uremia leads to a decrease in aortic elasticity that severely affects cardiovascular function [35,36]. In an experimental model of moderate uremia induced by 5/6 nephrectomy, it was shown how uremia induced systemic inflammation, modulated smooth muscle cell phenotype, increased aortic thickness, and decreased aortic constriction [37], whereas aortic stiffness due to uremia has been observed in patients with ESRD [38,39]. By deleting ILK before inducing ESRD in mice, both structural alterations and increases in the expression of fibrosis markers observed in animals on the adenine diet were prevented. In fact, ILK content in aortas correlated with the expression of these markers suggesting a direct relationship between them. On the other hand, although some studies have demonstrated antifibrotic effects of tamoxifen [40,41], the compound injected into mice to delete ILK, the likelihood that tamoxifen is responsible for the improvement of vascular alterations, rather than ILK, appears to be very low due to the short duration of treatment, the low dose administered to the animals and the time elapsed between administration and completion of the studies.

However, given that our study is based on the analysis of vascular damage secondary to a primary disease, CKD, and that ILK deletion in our model occurs globally throughout the organism, with the findings presented so far we cannot conclude that ILK blockade in the aortic tissue itself is responsible for the prevention of vascular damage observed in this model. The fact that ILK-deleted mice do not have as much impaired renal function, as we previously described [24], could explain why such damage secondary to the primary disease was not being caused. Moreover, the observed improvement in vascular morphological alterations could also be attributable to the attenuation of the adenine-induced elevation in arterial pressure in CKD-ILK mice.

To clarify this point, we performed ex vivo experiments with the aortas of WT and cKD-ILK mice exposed directly to the uremic toxins pCS and IS. Note that the toxin concentrations used in this study were selected to mimic advanced stages of CKD. In patients with CKD not undergoing dialysis, Eloot et al. analyzed the concentrations of IS and pCS, among other uremic toxins, in patients with CKD stages 2 to 5 and identified concentration ranges of 15–92 μg/mL for IS and 59–291 μg/mL for pCS [42]. In hemodialysis patients, the levels of these uremic toxins can reach up to 236 µg/mL for the IS and 105 µg/mL for pCS, respectively [43]. Moreover, in the adenine-induced CKD model, we have analyzed using UHPLC-MS/MS (ultra-high performance liquid chromatography-tandem mass spectrometry system) that there is a very significant increase in the levels of both uremic toxins concentrations in the mice plasma (Supplementary Table S1), from 2 weeks of administration of the diet. The presented results of the ex vivo study demonstrated the prevention of vascular damage in aortas from ILK-deletion mice under high uremic conditions, confirmed that ILK is not only important at the renal level [24], but also plays an essential role at the vascular level. Another way to have addressed this issue would have been a conditional tissue-specific deletion of ILK in the aorta of adult animals, so that the improvement at the renal level of the animals would not have affected the results. However, one study demonstrated that mice with ILK deletion in vascular smooth muscle cells survived to birth, but died in the perinatal period and exhibited multiple vascular pathologies, including aneurysmal dilatation of the aorta and patent ductus arteriosus [44].

Through in vitro studies, it has been observed that IS promotes the proliferation of vascular smooth muscle cells [45], that together migration and high production of ECM proteins, produces hyperplasia of the neointima, abnormalities in vascular repair and marked reduction in vascular lumen. Moreover, studies have attempted to elucidate the role of ILK specifically in vascular smooth muscle cells in both a physiological and pathophysiological context [34,46]. Indeed, several investigations have described ILK as a therapeutic target in processes such as vascular contraction [33], but only a few have identified its kinase activity as responsible for such processes [15,47]. Therefore, to further study whether ILK played a role in the vascular alterations observed in the in vivo CKD model and to identify the cell type responsible for them, we decided to perform in vitro experiments exposing vascular smooth muscle cells, the predominant cell type in the vascular wall, to the uremic toxins pCS and IS. Moreover, in these cells, we previously observed how the additive effect of the uremic toxins *p*-cresol and IS together induced the expression of ECM proteins and the profibrotic cytokine TGF-β1 [48], which could be the pathogenic mechanism explaining the vascular damage observed in vivo. The uremic toxins pCS and IS increased both ILK activity and the expression of fibrosis markers of vascular cells, but did not increase ILK expression, as we had observed in the in vivo model. This could mean that the increased ILK expression may be taking place in other cell types of aortic tissue, and not in this particular cell type, or that the stimulus of the uremic toxins pCS and IS is not the only one affecting ILK in these cells. Since the in vitro model did not exactly reproduce the in vivo and ex vivo findings regarding the increase in ILK expression levels, the relevance of the latter data appears to be limited. On the other hand, we have recently published, using the same animal model, that CKD induces an increase in ILK levels in peripheral blood mononuclear cells (PBMCs) [49]. We have also demonstrated that this increase enhances the adhesion and transmigration of these cells [23], and that ILK levels in PBMCs directly correlate with markers of inflammation in the aorta [49], which may provide further insight into processes occurring in the whole organism. Furthermore, Chen Wongworawat et al. recently identified distinct FCGR3A-high monocyte/macrophage subpopulations involved in kidney transplant rejection, reinforcing the emphasis on macrophage-driven pathology and supporting the broader idea that targeting specific innate immune pathways, such as ILK, may offer therapeutic benefits in CKD-related vascular complications [50]. Finally, we observed that silencing ILK in vascular smooth muscle cells, which blunted the uremia-induced increase in its kinase activity, prevented the expression of fibrosis markers, suggesting a direct relationship between them.

## 4. Materials and Methods

### 4.1. Animal Study Design

All procedures involving animals were previously approved by the Institutional Animal Care and Use Committee of the University of Alcalá and conformed to Directive 2010/63/EU of the European Parliament. Animals were housed in a pathogen-free and temperature-controlled room (22 °C ± 2 °C). Food and water were available ad libitum.

An inducible ILK knockdown mice (cKD-ILK) model, explained in prior publications [49], was used to perform in vivo and ex vivo experiments. Briefly, conditional inactivation of the ILK gene was accomplished by crossing C57Bl/6 mice homozygous for the floxed ILK allele (LOX mice) with homozygous mice carrying a tamoxifen-inducible CreER(T) recombinase gene, which express Cre under the control of the cytomegalovirus promoter (CRE mice). Tamoxifen (Sigma-Aldrich, Merck; St. Louis, MO, USA) was dissolved in a corn oil/ethanol (9:1) mixture. CRE-LOX mice (8-week-old), heterozygous for both transgenes, were injected intraperitoneally with 1.5 mg of tamoxifen once per day for 5 consecutive days to induce ILK depletion. Control animals were injected with the vehicle, a corn oil/ethanol (9:1) mixture. Three weeks after the injections, tail DNA was genotyped by PCR with primers that distinguish excised ILK gene (230 bp) or non-excised ILK (2100 bp): CCAGGTGGCAGAGGTAAGTA and CAAGGAATAAGGTGAGCTTCAGAA [24]. PCR DNA products were then analyzed by 1.5% agarose gel electrophoresis. Tamoxifen-treated CRE-LOX mice displaying successful depletion of ILK were termed cKD-ILK mice, and their control vehicle-treated CRE-LOX mice were termed wild-type (WT).

For the in vivo experiments, basal body weight and blood pressure were determined, and blood samples were extracted by incision of an inferior palpebral vein (0 week). CKD was induced in mice by feeding a diet containing 0.2% adenine (Sigma-Aldrich, Merck) as previously described [23,24]. After 2, 4, or 6 weeks, body weight and blood pressure of mice (14, 16, or 18 weeks old, respectively) were determined. Animals were then anesthetized, and blood and aortas were extracted. For plasma determinations, blood was collected in tubes with 0.1% EDTA as anticoagulant and plasma was separated by centrifugation at 3000 rpm for 15 min and stored at −80 °C until assayed. For the RT-qPCR assays, aortas were stored in RNAlater solution (Invitrogen, Thermo Fisher Scientific; Waltham, MA, USA) at −80 °C. For histological analysis, aortas were fixed in 4% *p*-formaldehyde, dehydrated and embedded in paraffin. For the ex vivo experiments, three weeks after tamoxifen injection, mice were anesthetized, and aortas of WT and cKD-ILK mice were collected in culture medium and treated ex vivo with both uremic toxins pCS (Tokio Chemical Industry; Tokio, Japan) and IS (Sigma-Aldrich, Merck) at high doses (226 µg/mL and 100 µg/mL, respectively) for 24 h. After incubation, aortas were stored in RNAlater solution at −80 °C for the RT-qPCR assays.

### 4.2. In Vitro Cultured Cells

The human aortic-vascular smooth muscle cells line HA-VSMC (Innoprot; Vizcaya, Spain) was maintained in DMEM culture medium supplemented with high glucose (4,5 g l^−1^), L-glutamine (20 mM) (Lonza; Basilea, Switzerland), antibiotics (penicillin, 100 U mL^−1^; streptomycin, 100 mg mL^−1^) (Fisher Scientific, Thermo Fisher Scientific, Waltham, MA, USA), and 10% fetal bovine serum (Sigma-Aldrich, Merck). Cells were cultured at 37 °C in a 5% CO_2_ atmosphere. Cells were used between passages 2 and 15.

For the experiments, cells were incubated with both uremic toxins, pCS and IS, at high doses (226 µg/mL and 100 µg/mL, respectively) and different times. pCS and IS were tested at different times and at high doses within the uremic range as previously described [23]. Briefly, pCS and IS were prepared in water at a stock concentration of 12.5 mg mL^−1^. The uremic solutes were diluted at least 1:1000 in culture medium to reach mean uremic concentrations. The uremic solutes were compared with their control (water).

### 4.3. Clinical and Biochemical Assessments

Body weights were recorded at baseline (0 week) and at the end of the adenine treatments (2, 4 or 6 weeks). Blood pressure was measured in conscious mice placed on a heated platform (Hatteras Instruments, Cary, NC, USA) using a tail-cuff sphygmomanometer (LE 5001 Pressure Meter, Letica Scientific Instruments, Hospitalet, Spain). Blood pressure was considered as the mean of at least twenty consecutive valid measurements.

To assess renal function, plasma creatinine (700460; Cayman Chemical; Ann Arbor, MI, USA) and urea nitrogen (EIABUN; Invitrogen, Thermo Fisher Scientific; Waltham, MA, USA) were measured using colorimetric assay kits, according to the manufacturer’s instructions. The spectrophotometric measurements were performed in a Victor X4 Multilabel Plate Reader (PerkinElmer, Waltham, MA, USA) at a wavelength of 490 nm (plasma creatinine) and 450 nm (plasma urea nitrogen).

### 4.4. Reverse Transcription– Quantitative Polymerase Chain Reaction (RT-qPCR)

Total RNA of each sample was extracted with TRIzol (Invitrogen, Thermo Fisher Scientific), transcribed into cDNA with a High-Capacity cDNA Reverse Transcription Kit (Applied Biosystems, Thermo Fisher Scientific, Waltham, MA, USA) and RT-qPCR analysis was performed in a 7500-qPCR thermocycler. Non-excised ILK mRNA levels were measured in mice aortas by RT-qPCR with SYBR Green Master Mix to verify that ILK depletion had also occurred in these cells and tissues [23]. Primers GGGCTCTTGTGAGCTTCTGT and GAGTGGTCCCCTTCCAGAAT were designed to recognize the cDNA sequence between exons within floxed areas 6 and 7 [23,24]. For the study of gene expression in mice aortas, RT-qPCR with SYBR Green Master Mix were performed using primers designed in the PUBMED Gene Database: collagen type I (COL I), 5′-TCCTGGCAACAAAGGAGACA-3′ (forward) and 5′-GGGCTCCTGGTTTTCCTTCT-3′ (reverse); fibronectin (FN), 5′-TGAGCGCCCTAAAGATTCCA-3′ (forward) and 5′-TAGCCACCAGTCTCATGTGC-3′ (reverse); TGF-β1, 5′-TTGCTTCAGCTCCACAGAGA-3′ (forward) and 5′-TGGTTGTAGAGGGCAAGGAC-3′ (reverse); β-actin, 5′-GACGGCCAGGTCATCACTAT-3′ (forward) and 5′-CTTCTGCATCCTGTCAGCAA-3′ (reverse). TaqMan gene expression assays were used to quantify COL I (Hs00164004_m1), FN (Hs01549976_m1), TGF-β1 (Hs00998133_m1), and β-actin (Hs01060665_g1) in HA-VSMC. Amplification values were normalized to endogenous β-actin and relative quantification of gene expression was determined with 2^−ΔΔCT^ method [23,24].

### 4.5. Histology

Aortas processed in paraffin were de-paraffined in xylene and then hydrated in descending order of ethanol dilutions to finally be stained with Hematoxylin and Eosin Staining kit (Casa Álvarez: Madrid, Spain) or Picro Sirius Red Stain kit (Quimigen; Madrid, Spain) according to the manufacturer’s instructions. After that, they were dehydrated and mounted with DPX solution (PanReac AppliChem; Barcelona, Spain) to be observed with a microscope. Pictures were obtained with 20× and 40× magnification. Morphological alterations were determined in Hematoxylin-eosin staining by measuring the thickness of the media, the radius of the lumen and the ratio between the thickness of the media and the diameter of the lumen using Image J software 2.6. Aorta fibrosis was assessed by measuring the intensities of Sirius red by using Image Pro-Plus software 5.1 (https://www.mediacy.com/imageproplus, accessed on 23 November 2025) (Rockville, MD, USA).

### 4.6. Western Blot

Cells were homogenized in lysis buffer (10 mM Tris–HCl, pH 7.6; 1% Triton X-100; 1 mM EDTA; 0.1% sodium deoxycholate) supplemented with protease and phosphatase inhibitor cocktails (Roche, Basel, Switzerland). Protein concentrations were determined, and equal amounts of protein (20–40 µg) were separated on SDS–polyacrylamide gels and transferred to PVDF membranes (Bio-Rad, Richmond, CA, USA). After membranes were blocked, they were incubated with primary and secondary antibodies (Sigma-Aldrich, Merck, or Dako, Glostrup, Denmark). Primary antibodies used were against AKT, P-AKT (Ser473), COL I, GSK-3β, P-GSK-3β (Ser9), ILK (Cell Signaling Technology, Danvers, MA, USA). Membranes were reblotted with anti-GAPDH antibody (Sigma-Aldrich, Merck) to normalize protein levels. Immunoblots were detected by chemiluminescence (Pierce ECL Western Blotting Substrate; Thermo Scientific, Thermo Fisher Scientific) imaged with ImageQuant LAS 500 System (General Electric Healthcare, Uppsala, Sweden). Densitometries were measured using ImageJ software 2.6.

### 4.7. siRNA Transfection

To deplete expression of ILK protein by specific siRNAs, HA-VSMC were cultured with fetal bovine serum-free medium with 10 nM ILK-specific siRNA (Bionova Científica; Barcelona, Spain), or silencer-negative control (Scrambled RNA) (Invitrogen, Thermo Fisher Scientific) using Lipofectamin 2000 (Invitrogen, Thermo Fisher Scientific) transfection reagent during 6 h. After incubation at 37 °C in a 5% CO_2_ atmosphere with the RNA complex, 1 mL of medium containing FBS was added over night and then cells were treated as indicated.

### 4.8. Immunostaining Assay

For immunostaining against COL I, cells were treated as indicated and fixed with 4% *p*-formaldehyde, and permeabilized with 0.05% of Triton X-100. After blockade, HA-VSMC were stained with primary antibody against COL I (Cell Signaling Technology) and secondary antibodies (Invitrogen, Thermo Fisher Scientific). The cells were stained with Hoechst 33342 (Invitrogen, Thermo Fisher Scientific), and the coverslips were mounted with Prolong Gold antifade (Invitrogen, Thermo Fisher Scientific). The samples were analyzed using a LEICA TCS-SP5 confocal microscope (Leica Microsystems, Wetzlar, Germany). Four sequential confocal optical sections of randomly chosen fields were analyzed, and imaging analysis was performed by ImageJ software 2.6.

### 4.9. Statistical Analysis

All the data were analyzed with the GraphPad Prism 6 (GraphPad Prism Software Inc.; San Diego, CA, USA). The results are expressed as the mean ± SEM. As the number of animals or samples in the different experiments was never over 10, non-parametric statistics were used for comparisons, applying the Kruskal–Wallis test with Mann–Whitney post-test (non-paired data) or the Friedman test with Wilcoxon post-test (paired data). In both cases, Bonferroni correction was used. Correlation analysis was performed using linear regression for each genotype (combining WT Control, cKD-ILK Control, WT Adenine, and cKD-ILK Adenine treatments) and plotted on the same graph. A *p* value < 0.05 was considered statistically significant.

Additional methods are described in Supplementary Material.

## 5. Conclusions

This study confirms the involvement of ILK in the pathogenesis of vascular problems associated with CKD and demonstrates that ILK blockade prevents these alterations from the early stages of CKD to ESRD. In addition, its kinase function could be responsible for activating the signaling pathways responsible for the production of vascular fibrosis in vascular smooth muscle cells. Therefore, the controlled blockade of this protein, either by reducing its synthesis or its activity, could prevent or improve vascular lesions that develop as a consequence of the accumulation of uremic toxins.

## Figures and Tables

**Table 1:** Renal function parameters of wild-type (WT) and ILK conditional-knockdown (cKD-ILK) mice fed a standard (Control) or an adenine-rich (Adenine) diet for 0, 2, 4 or 6 weeks.

	Diet Weeks	0 Weeks	2 Weeks	4 Weeks	6 Weeks
Plasma Parameters					
Urea Nitrogen (mg/dL)					
WT Control		23.8 ± 2.8	24.0 ± 5.5	24.4 ± 5.8	25.3 ± 3.1
cKD-ILK Control		24.3 ± 3.6	23.8 ± 2.7	24.0 ± 6.1	22.8 ± 1.2
WT Adenine		27.1 ± 3.4	82.2 ± 20.1 *^$^	104.3 ± 15.1 *^$^	119.4 ± 9.1 *^$^
cKD-ILK Adenine		24.2 ± 1.9	46.8 ± 10.2 *^$#^	51.6 ± 15.8 *^$#^	55.4 ± 10.8 *^$#^
Creatinine (mg/dL)					
WT Control		0.26 ± 0.05	0.27 ± 0.07	0.30 ± 0.07	0.25 ± 0.01
cKD-ILK Control		0.27 ± 0.06	0.28 ± 0.06	0.31 ± 0.05	0.29 ± 0.04
WT Adenine		0.38 ± 0.05	0.64 ± 0.11 *^$^	0.73 ± 0.10 *^$^	0.87 ± 0.06 *^$^
cKD-ILK Adenine		0.29 ± 0.04	0.43 ± 0.07 *^$#^	0.48 ± 0.08 *^$#^	0.49 ± 0.05 *^$#^

Renal function was assessed by measuring plasma creatinine and urea nitrogen concentrations of WT and cKD-ILK mice fed a standard or an adenine-rich diet for 0, 2, 4 or 6 weeks. Results are shown as mean ± SEM. * *p* < 0.05 vs. WT Control at the same time; ^$^ *p* < 0.05 vs. 0 weeks; ^#^ *p* < 0.05 vs. WT Adenine at the same time. n = 3–9 animals/group.

**Table 2:** Parameters of wild-type (WT) and ILK conditional-knockdown (cKD-ILK) mice fed a standard (Control) or an adenine-rich (Adenine) diet for 0, 2, 4 or 6 weeks.

	Diet Weeks	0 Weeks	2 Weeks	4 Weeks	6 Weeks
Parameters					
Body weight (g)					
WT Control		27.2 ± 1.9	30.7 ± 0.9	30.3 ± 3.6	31.2 ± 3.6
cKD-ILK Control		26.6 ± 2.2	28.8 ± 2.9	29.2 ± 3.8	29.4 ± 3.1
WT Adenine		27.4 ± 3.3	21.7 ± 2.7 *^$^	22.1 ± 2.0 *^$^	21.9 ± 2.5 *^$^
cKD-ILK Adenine		27.0 ± 1.3	23.2 ± 1.5 *^$^	23.5 ± 1.9 *^$^	22.8 ± 2.1 *^$^
Mean arterial pressure (mmHg)					
WT Control		89.2 ± 0.7	88.5 ± 0.6	90.3 ± 1.1	90.5 ± 2.1
cKD-ILK Control		89.1 ± 0.6	89.0 ± 1.5	89.5 ± 0.9	89.8 ± 1.2
WT Adenine		88.3 ± 1.7	93.5 ± 4.9	112.5 ± 4.9 *^$^	127.5 ± 9.2 *^$^
cKD-ILK Adenine		88.5 ± 0.8	90.3 ± 1.8	91.2 ± 2.6 ^#^	94.6 ± 4.2 *^#^

Body weight (g) and mean arterial pressures (mmHg) of WT and cKD-ILK mice fed a standard or an adenine-rich diet for 0, 2, 4 or 6 weeks. Results are shown as mean ± SEM. * *p* < 0.05 vs. WT Control at the same time; ^$^ *p* < 0.05 vs. 0 weeks; ^#^ *p* < 0.05 vs. WT Adenine at the same time. n = 3–9 animals/group.

## Data Availability

The original contributions presented in this study are included in the article/Supplementary Material. Further inquiries can be directed to the corresponding author.

## Supplementary Materials

The following supporting information can be downloaded at: https://www.mdpi.com/article/10.3390/ijms27010215/s1.

## Abbreviations

The following abbreviations are used in this manuscript:

CKD	Chronic kidney disease
cKD-ILK	ILK conditional-knockdown
COL I	Collagen type I
CT	Control
CVD	Cardiovascular disease
ECM	Extracellular matrix
ESRD	End-stage renal disease
FN	Fibronectin
HA-VSMC	Human aortic-vascular smooth muscle cells
ILK	Integrin-linked kinase
IS	Indoxyl sulfate
PBMCs	Peripheral blood mononuclear cells
pCS	*p*-cresyl sulfate
RT-qPCR	Reverse transcription-quantitative polymerase chain reaction
WT	Wild-type
