# Supplementary material for: ILK Deletion Protects Against Chronic Kidney Disease-Associated Vascular Damage
Source: Int J Mol Sci. 2025 Dec 24;27(1):215. doi: 10.3390/ijms27010215 (PMC12785976; doi:10.3390/ijms27010215)
Supplement: Supplementary file 1 [file ijms-27-00215-s001.zip › ijms-4032367-supplementary.pdf]

SUPPLEMENTARY MATERIAL

Article

ILK deletion protects against chronic kidney disease-associated vascular damage

Sofía Campillo [1,2,3,4,5,*], Elena Gutiérrez-Calabrés [1,2,3,4,5], Susana García-Miranda [1,2,3,4,5], Mercedes Griera [1,2,3,4,5], Sergio de Frutos [1,†], Diego Rodríguez-Puyol 2[,3,4,5,6,7,†] and Laura Calleros [1,2,3,4,5,†]

1 Physiology Unit, Department of Systems Biology, Universidad de Alcalá, 28871 Alcalá de Henares, Madrid, Spain; elena.gutierrez@uah.es (E.G.-C.); susana.garciam@uah.es (S.G.-M.); mercedes.griera@uah.es (M.G.); sergio.frutos@uah.es (S.d.F.); laura.calleros@uah.es (L.C.)
2 Fundación Renal Española, 28003 Madrid, Spain; diego.rodriguez@uah.es
3 Instituto Ramón y Cajal de Investigación Sanitaria (IRYCIS), 28034 Madrid, Spain
4 INNOREN-CM, 28049 Madrid, Spain
5 RICORS 2040 RENAL, Instituto de Salud Carlos III, 28029 Madrid, Spain
6 Department of Medicine and Medical Specialties, Universidad de Alcalá, 28871 Alcalá de Henares, Madrid, Spain
7 Biomedical Research Foundation and Nephrology Unit, Hospital Universitario Príncipe de Asturias, 28805 Alcalá de Henares, Madrid, Spain
* Correspondence: sofia.campillo@uah.es; Tel.: +34-918854521
† These authors shared senior direction of this work.

1. Supplementary Methods

1.1. Cell viability assays

The effect of pCS and IS on cell viability was determined by propidium iodide exclusion in the flow cytometer MACSQuant Analyzer 10 (Miltenyi Biotec, Bergisch Gladbach, Germany). Briefly, HA-VSMC cells were seeded on six-well culture plates until 60-80% confluence and incubated with high doses of pCS and IS (226 μg/ml and 100 μg/ml, respectively) at different times. After incubation, cells were centrifuged and resuspended in 0,5 ml PBS, and 5 μl of propidium iodide (Sigma-Aldrich, Merck) were added to each condition. The percentage of cells excluding propidium iodide was determined by considering the control condition (no treatment with uremic toxins) as 100% viability and normalizing all other conditions with respect to that.

1.2. Immunostaining assay

For immunostaining against ILK, cells were treated and fixed with 4% PFA, and permeabilized with 0,05% of Triton X-100. After blockade, HA-VSMC were stained with primary antibody against ILK (Abcam, Cambridge, UK) and secondary antibodies (Invitrogen, Thermo Fisher Scientific). The cells were stained with Hoechst 33342 (Invitrogen, Thermo Fisher Scientific), and the coverslips were mounted with Prolong Gold antifade (Invitrogen, Thermo Fisher Scientific). The samples were analyzed using a LEICA TCS-SP5 confocal microscope (Leica Microsystems, Wetzlar, Germany). Four sequential confocal optical sections of randomly chosen fields were analyzed, and imaging analysis was performed by ImageJ software 2.6.

1.3. Determination of uremic toxins in plasma

The determination of total IS and pCS concentrations was performed at the Metabolomics and Bioanalysis Center (CEMBIO) of Universidad San Pablo-CEU using ultra-high-performance liquid chromatography–tandem mass spectrometry (UHPLC-MS/MS). Plasma samples were treated using protein precipitation with acetonitrile containing stable isotope-labeled pCS-D7 as an internal standard. For this analysis, 5 μl of sample was injected, and chromatographic separation was achieved with an Acquity UPLC® BEH C18 column (1.7 μm, 2.1 mm × 100 mm) and a Vanguard Acquity UPLC® BEH C18 pre-column (2.1 × 5 mm), maintained at 30 °C in the UHPLC system oven (1290 Infinity, Agilent Technologies, Waldbronn, Germany). The mobile phase consisted of 0.1% formic acid (v/v) in water for phase A and 0.1% formic acid (v/v) in acetonitrile for phase B. Analyte concentrations were calculated from the calibration curve of ion ratios between the analytes and the internal standard.

2. Supplementary Figures and Figure Legends

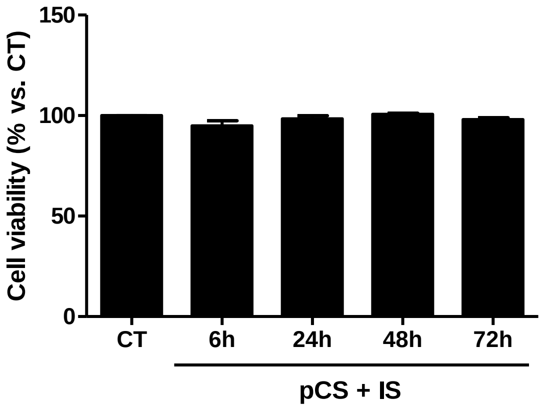

Supplementary Figure S1. *p*-cresyl sulphate (pCS) and indoxyl sulphate (IS) do not affect viability of HA-VSMC cells. HA-VSMC cells were incubated with a combination of pCS and IS at high doses (226 µg/ml and 100 µg/ml, respectively) for 6, 24, 48, and 72 hours. After incubation with the uremic toxins, cell viability was determined by propidium iodide exclusion. Results were expressed as a percentage of the number of untreated control cells (CT). All values are represented as mean ± SEM of 3 independent experiments.

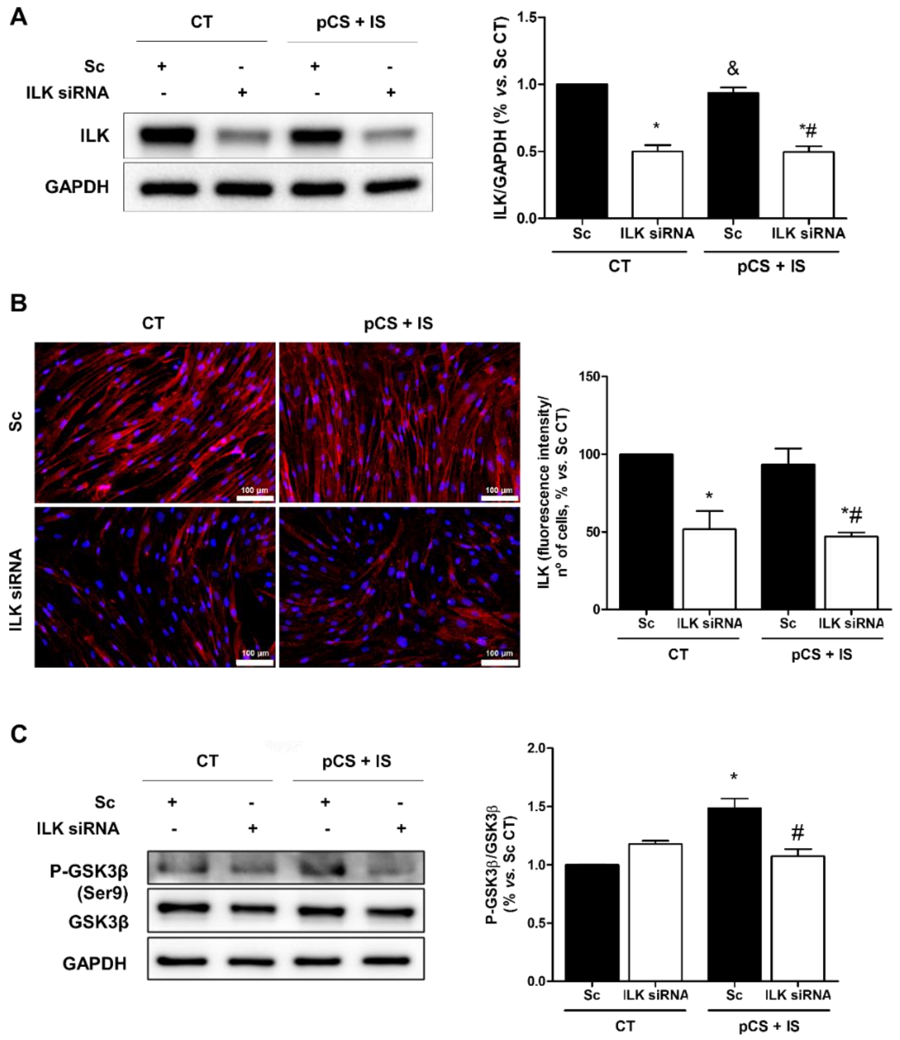

Supplementary Figure S2. ***p*-cresyl sulfate (pCS) and indoxyl sulfate (IS) increase GSK-3β phosphorylation levels through ILK in HA-VSMC cells.** HA-VSMC cells were transfected with scrambled RNA (Sc, black bars) or were depleted of ILK with specific siRNA (ILK siRNA, white bars) and were incubated with a combination of pCS and IS at high doses (226 µg/ml and 100 µg/ml, respectively) for 6 h. After incubation with the uremic toxins, ILK expression (**A**) and phosphorylation levels of GSK-3β at serine 9 (P-GSK-3β) (**C**) were measured by western blot. Representative western blots of ILK and P-GSK-3β are shown. GAPDH and GSK-3β were used as the endogenous controls, respectively. The bars represent the normalized densitometric values of the blots against the endogenous control values. (**B**) Prior to transfection, cells were seeded on coverslips and, after incubation with the uremic toxins, cells were labeled with an anti-ILK antibody (red) and nuclei with Hoechst 33342 (blue). Fluorescence intensity was determined by fluorescence microscopy. Images from a representative experiment are shown. Scale bar: 100 µm. The bar graph represents the average percentages of ILK fluorescence intensity normalized with respect to the number of cells in each image. Results were expressed as a percentage with respect to the Sc untreated control (CT). All values are presented as the mean ± SEM of 3 or 4 independent experiments. *p<0.05 *vs.* Sc CT; &p<0.05 *vs.* ILK siRNA CT; #p<0.05 *vs.* Sc (pCS+IS).

Supplementary Table S1. Uremic toxins plasma levels of wild-type (WT) and ILK conditional-knockdown (cKD-ILK) mice fed a standard (Control) or an adenine-rich (Adenine) diet for 0, 2, 4 or 6 weeks.

DIET WEEKS / UREMIC TOXINS	0 WEEKS	2 WEEKS	4 WEEKS	6 WEEKS
***p*-CRESYL SULFATE (pCS) (μg/ml)**				
**WT Control**	0.7 ± 0.3	0.5 ± 0.1	0.9 ± 0.2	0.9 ± 0.1
**cKD-ILK Control**	1.2 ± 0.6	1.0 ± 0.2	0.6 ± 0.1	0.8 ± 0.2
**WT Adenine**	0.4 ± 0.1	5.3 ± 1.6*$	9.2 ± 2.4*$	13.7 ± 1.4*$
**cKD-ILK Adenine**	0.6 ± 0.2	3.1 ± 0.8*$	7.2 ± 2.0*$	10.7 ± 1.7*$
**INDOXYL SULFATE (IS) (μg/ml)**				
**WT Control**	1.9 ± 0.6	2.3 ± 0.9	1.3 ± 1.0	1.3 ± 0.8
**cKD-ILK Control**	1.6 ± 0.4	1.6 ± 0.7	1.7 ± 0.4	1.4 ± 0.6
**WT Adenine**	1.0 ± 0.5	5.9 ± 1.3*$	11.0 ± 0.9*$	25.2 ± 2.9*$
**cKD-ILK Adenine**	1.5 ± 0.1	4.2 ± 0.5*$	9.2 ± 1.1*$	19.3 ± 3.2*$

Total pCS and IS plasma levels of WT and cKD-ILK mice fed a standard or an adenine-rich diet for 0, 2, 4 or 6 weeks were analyzed by UHPLC-MS/MS. Results are shown as mean ± SEM. *p<0.05 vs. WT Control at the same time; $p<0.05 vs. 0 weeks. n=5-8 animals/group.
